# The location of the permanent mandibular canine as identified in orthopantomograms from children younger than 5 years of age: a case series study

**DOI:** 10.1007/s40368-023-00825-y

**Published:** 2023-08-24

**Authors:** I. Kjær, M. Svanholt, P. Svanholt

**Affiliations:** 1 Department of Odontology. Faculty of Health and Medical Sciences, University of Copenhagen, Copenhagen, Denmark; 2 Section of Orthodontics, Department of Odontology, Faculty of Health and Medical Sciences, Copenhagen, Denmark; 3 Copenhagen Municipal Clinic of Orthodontics, Copenhagen, Denmark; 4 Guldborgsund Municipal Clinic of Orthodontics, Nykøbing Falster, Denmark

**Keywords:** Permanent canine, Mandible, Eruption, Orthopantomogram, Primary dentition

## Abstract

**Aim:**

The aim of this case series study is to describe where the crowns of the permanent mandibular canines are located in early childhood in relation to the preceding primary canines.

**Materials and methods:**

From a sample of 31 orthopantomograms from children, younger than 5 years of age, the location of the mandibular canines was analysed by visual inspection. The radiographs were taken due to different deviations in the dentition and forwarded during a period of 28 years for elucidating different diagnostic questions. From an embryological point of view, the primary canine was considered as a stable structure in the jawbone. A longitudinal axis through the primary canine was named the *canine axis.* The initial site of the permanent crown was the site, where the permanent lamina “placed” the tooth bud for final development. A normal site was determined when the *canine axis* passed though the permanent crown and a pathological site was determined when this did not happen.

**Results:**

Normal sites for the permanent crowns were observed in 19 cases and abnormal sites in 8 cases. In four cases, the interrelationship between the *canine axis* and the permanent crown could not be decided. The study demonstrated that the tooth germ for the permanent canine can start initial formation misplaced distally or mesially to the preceding primary tooth.

**Conclusion:**

The study demonstrates different malpositions of the initial crowns of the permanent mandibular canines in children younger than 5 years of age. The results are considered of importance for future understanding of the aetiology behind ectopic mandibular canines.

## Introduction

It has been mentioned that the aetiology behind horizontally positioned permanent mandibular canines might be a malposition of the initial crown anlage before root formation early in childhood (Joshi 2001). However, histological or radiologically studies proving this hypothesis have never been carried out. The problem is that the ectopic horizontally positioned permanent canine is usually diagnosed at puberty, but the position of the initial crown anlage can only be diagnosed before age 5—an age where there is no clinical indication of canine ectopia or justification for its radiographic investigation. This explains the severe lack of longitudinal series of radiographs demonstrating development of permanent mandibular canine ectopia.

The purpose of the present study is to focus on where the crowns of the developing mandibular canines are located in early childhood, in relation to the preceding primary canines. Furthermore, the goal is to suggest what might be considered a normal site for location of the permanent mandibular canine and what might be considered a pathological site for location.

A similar study demonstrating initial positions of the permanent crown anlage of the mandibular canine, which is necessary for understanding the aetiology behind canine ectopia, seems to be lacking in the literature.

### Mandible development and growth pattern in the canine region

The primary mandibular canine bordering the incisors and premolars has a stable position in the dental arch (Kjær 2017). The bone in the canine area is the first bony tissue formed in the prenatal mandible (Kjær 1975, 1989, 2017). The prenatal mandibular canine develops in a separate alveolus in the growing alveolar process (Kjær and Bagheri 1999). Space for the developing mandibular incisors, located in a shared alveolus, is gained from early growth in the symphysis menti region and growth of the alveolar process (Kjær 1975, 2017). Space for the mandibular primary molars and premolars is obtained from the posterior elongation of the mandible during growth combined with growth in the alveolar process (Bjørk and Skieller 1983). The stable primary canine position means that the primary canine and its successor, the permanent mandibular canine, do not migrate during development in the mandible, neither in the mesial direction nor in the distal direction. The vertical alveolar bone growth in the mandible creates space for the canines.

### Permanent canines and premolars: comparisons

#### Permanent canines

**Fig. 1:**
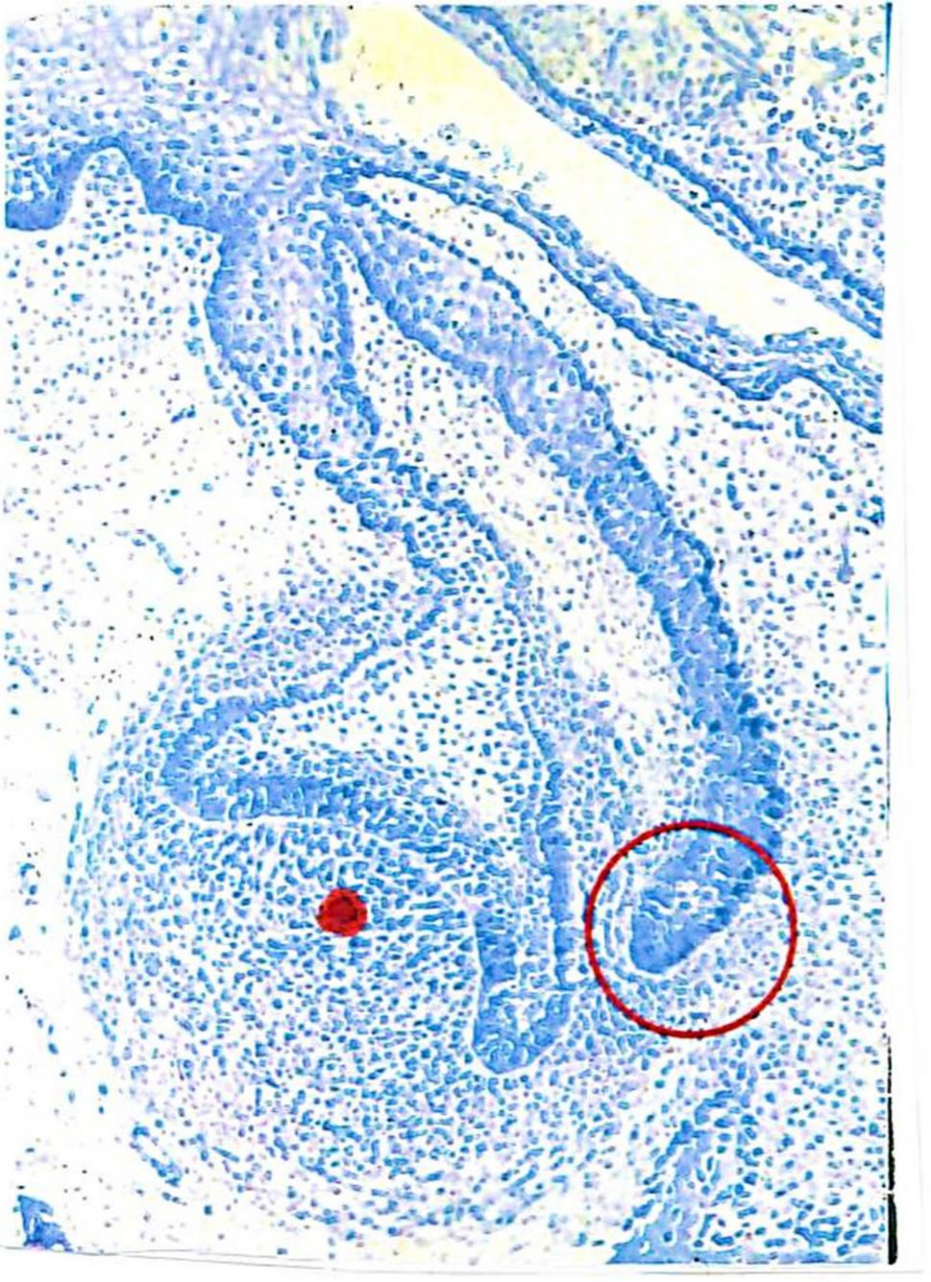
Histological section demonstrating an early tooth bud from a primary tooth and the extended dental lamina forming its permanent successor, marked by a red circle. The red dot underneath the primary tooth bud indicates the normal final position of the permanent tooth

The formation of the permanent canine starts from a tooth bud growing out from the primary dental lamina (Kjær1980), demonstrated in Fig. 1. This permanent tooth bud marked by a red circle, is initially located laterally to the primary tooth anlage.

From this lateral position, the permanent tooth bud normally finds its final position underneath the primary tooth anlage. This seems to happen during onset of eruptive movements of the primary tooth (Kjær et al 2008). Accordingly, the primary and permanent canines will share the same vertical axis during normal development. If this normal change in position of the permanent tooth bud does not occur, then the crown of the permanent canine will develop in an ectopic position and, as a consequence, develop and erupt ectopically (Kjær 2021).

#### Premolars

In a recent study, abnormal premolar eruption was classified, based on the insight from the same normal histological development illustrated in Fig. 1 (Kjær 2021). This study demonstrated that ectopic premolars could be located both posteriorly and anteriorly to the primary molars. Also abnormal upside-down position of premolars was recorded, with the root of the premolar between the primary molar roots and the crown of the premolar in the direction opposite to the primary crown (upside-down) (Kjær 2021). This study also demonstrated that normal shedding and ankyloses of the primary molars influenced the ectopic positions.

The background for comparing permanent canine position with premolar position is that the mandibular premolars and the mandibular canine belong to the same developmental field with the same innervation (Kjær et al 1994; Kjær 1995, 2014, 2017). It has been described that the postnatal stages of tooth development are closely interrelated within the canine/premolar field (Svanholt and Kjær 2008). Furthermore, the eruption times of the mandibular permanent canine and first and second premolars within the developmental field are closely identical and different from eruption times in other fields (Parner et al. 2002). These circumstances make it obvious to compare the initial positions of the mandibular premolars with the initial positions of the mandibular permanent canines.

## Materials and methods

### Materials

Radiographic material, particularly orthopantomograms and profile radiographs, from 3800 clinical cases of dental and craniofacial disorders have been received from paedodontic and orthodontic clinics, specifically from Denmark and other Nordic Countries. The material has been forwarded during a period of 28 years (1992–2020) for elucidating different diagnostic questions and for treatment planning.

#### Actual sample size

Amongst the orthopantomograms received, only 31 orthopantomograms were taken from children before age 5. Of these orthopantomograms, 2 were taken from children 3 years and 11 months old and 11 were taken from children between 4 years, 0 months and 4 years, 6 months. Furthermore, 18 orthopantomograms were taken from children between 4 years 7 months and 5 years 0 month of age. This is the material investigated in the present study. The indications for taking these radiographs were clinically observed eruption problems, specifically in the primary molars or for observations for other dental deviations, such as multiple agenesis, trauma problems, and jaw asymmetry. None of these 31 orthopantomograms were taken for prediction or prevention of eruption problems in the permanent dentition.

### Methods

Each orthopantomogram was analysed by visual inspection for presence of mandibular primary canines and for maturity stage and location (initial site) of permanent mandibular canines.

#### Primary canines and canine axis

Primary mandibular canines were present in all orthopantomograms. A longitudinal or length axis was constructed through the primary canine. This axis is named the canine axis. This axis is stable and of importance for defining location of the permanent canine.

#### Permanent canines

The maturity stage of the permanent canine was recorded as “crown under development”, “crown finished formation” and in a few cases “onset of root formation”.

#### Initial site

*Initial site* is defined as the site where the permanent lamina “placed” the tooth bud for final development of the permanent crown before root development.

#### Normal site

*Normal site* was observed when the canine axis from the primary canine passed through the entire crown of the permanent canine.

#### Abnormal site

*Abnormal site* was observed when the canine axis did not pass through the entire permanent crown, but only touched the uppermost crown contour or if the canine axis did not touch the permanent canine at all.

## Results

### Canine axis

In four cases, it was difficult to decide how the canine axis passed the crown of the permanent canine.

### Normal site

**Fig. 2:**
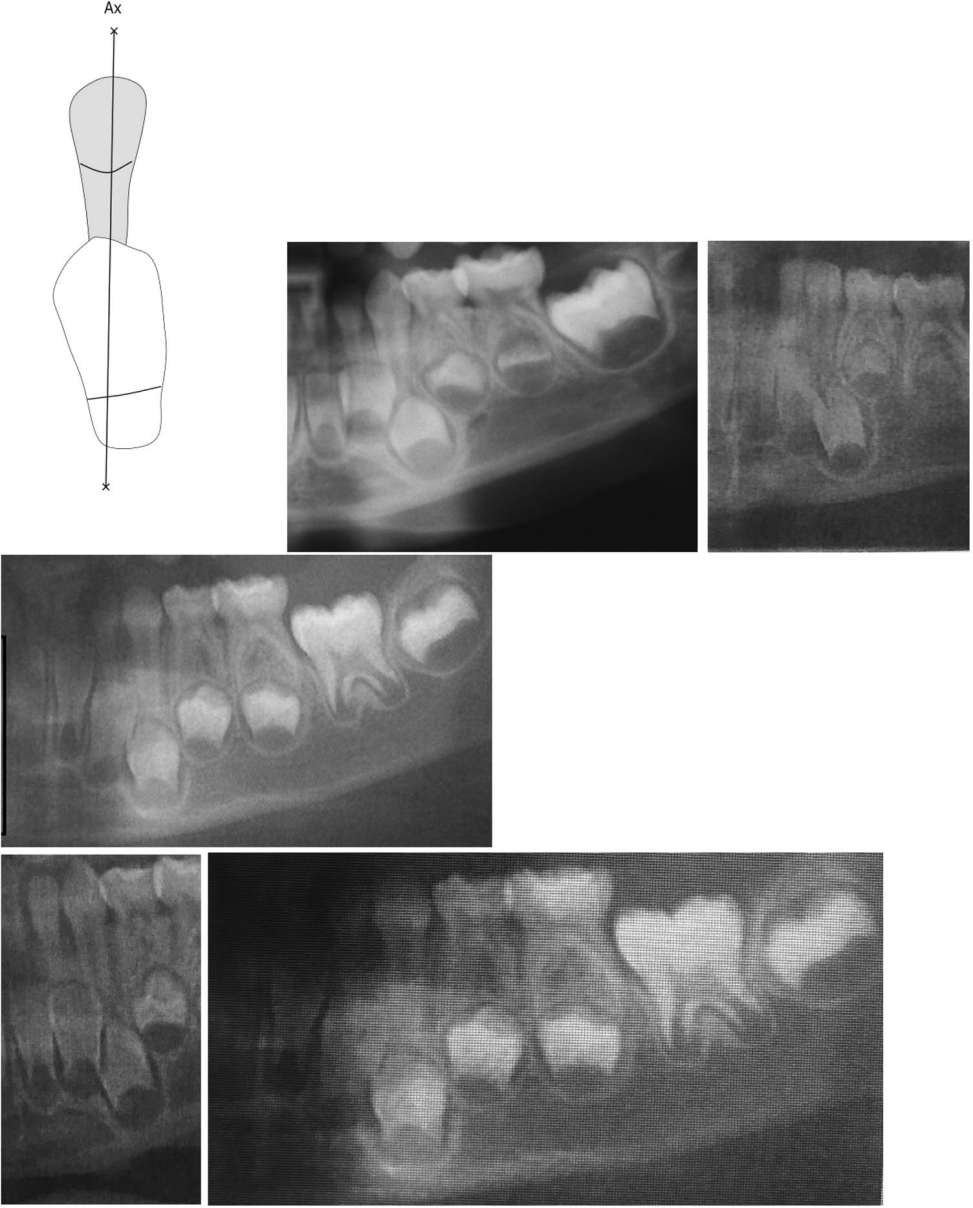
Normal interrelationships between the left-sided mandibular primary canine and the crown of its permanent successor. The drawing indicates the mandibular primary canine (hatched) marked by a longitudinal axis, Ax. The crown of the permanent canine is located below the primary canine and the canine axis is observed within the contour of the permanent crown. The five radiographs are from canine regions observed in orthopantomograms from children, younger than 5 years of age. The interrelationship between the primary and permanent canines is as indicated in the drawing

Normal site for the permanent tooth crown was a location underneath the primary canine root. This was observed in 19 cases. Five of these cases are demonstrated in Fig. 2.

### Abnormal site

**Fig. 3:**
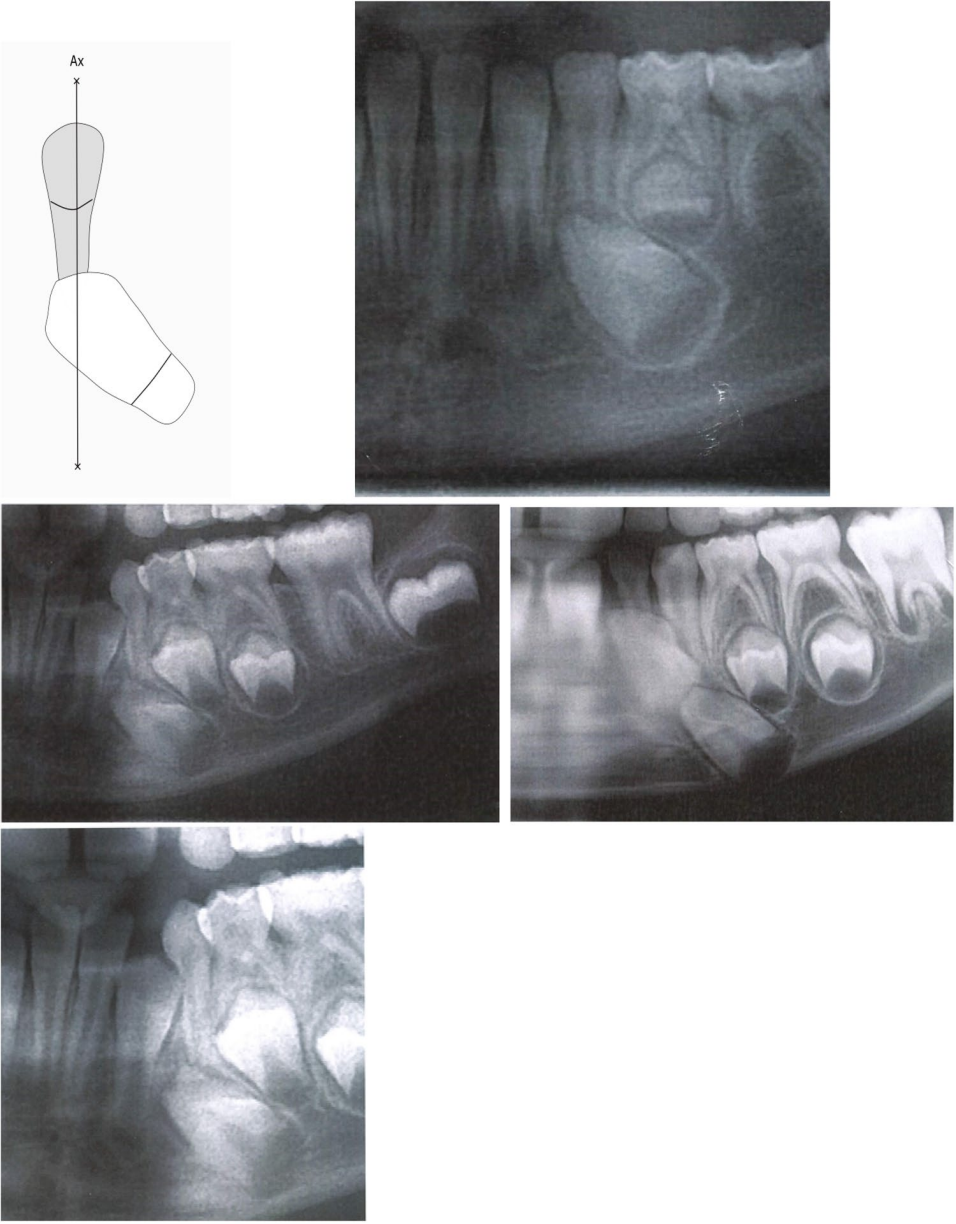
Abnormal interrelationships between the left-sided mandibular primary canine and the crown of its permanent successor. The drawing indicates the primary canine and its axis, marked Ax. The axis of the primary canine touches the most coronal part of the crown of the permanent canine. The permanent canine is located posteriorly compared to the canine axis. The four radiographs are from canine regions in orthopantomograms from children, younger than 5 years of age. Notice that the canine axis only pass through the uppermost part of the permanent crown. Furthermore, the permanent crowns are severely tilted, compared to normal location, observed in Fig. 2

**Fig. 4:**
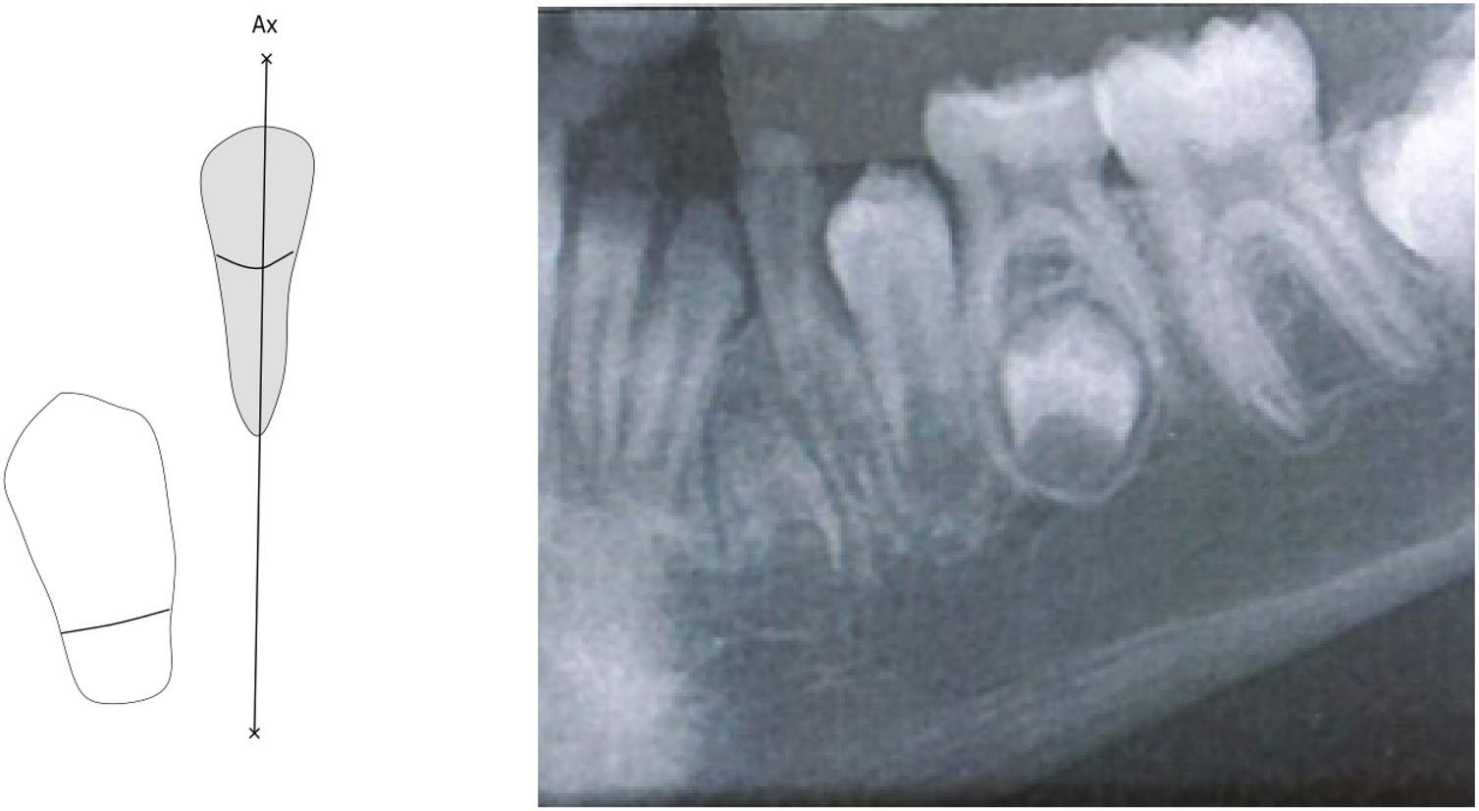
Abnormal interrelationships between the left-sided mandibular primary canine and the crown of its permanent successor. The drawing indicates the primary canine and its axis, marked Ax. The axis of the primary canine does not touch the crown of the permanent canine. The permanent canine is located mesially compared to the canine axis. The radiograph is from a canine region in an orthopantomogram from a child, 4 years and 11 months of age. Notice that the permanent anlage is located between the primary canine and the lateral incisor. Furthermore, there is a disagreement between the information of chronological age and the dental maturity registered

Abnormal site for the permanent tooth crown was observed in eight cases. A distal position of the permanent crown (often tilted) was observed in seven cases. Four cases are illustrated in Fig. 3. Mesial position of the permanent crown compared to the canine axis was observed in one case, illustrated in Fig. 4.

**Fig. 5:**
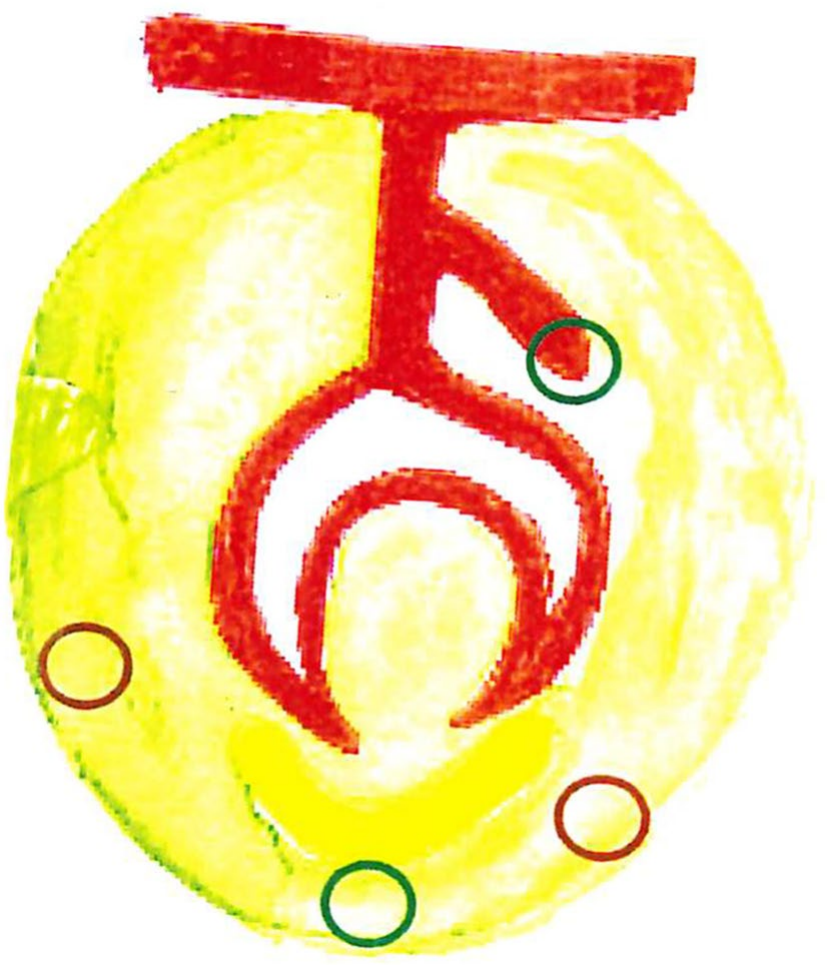
Schematical drawing of a primary tooth germ with an extended dental lamina for the permanent canine, marked by a green circle. The drawing indicates that the green circle below the primary tooth bud is the normal final location for the permanent canine. The two brown circles indicate pathological locations for the permanent canines. The right brown circle indicates a pathological distal location, and the left brown circle indicates a pathological mesial location of the permanent canine

The study demonstrated that the tooth germ for the permanent canine can start initial formation misplaced distally or mesially to the presiding primary tooth, as illustrated in Fig. 5.

## Discussion

The study design in this present study involved visual inspection of orthopantomograms, which includes uncertainty. Also the orientation of small children in a cephalostat can be a problem influencing the radiation and quality of the radiograph. Furthermore, the radiographs available for the present study did not represent normally developing dentitions as there would not have been indications for taking radiographs in such cases. Some orthopantomograms were from children having inborn deviations in the dentitions and some were from children having acquired deviations. This results in difficulties in conclusions concerning aetiology. Accordingly, the material only allow conclusions concerning the ability for a dental lamina from the primary canine for misplacing a germ forming the permanent canine crown. This aspect has seemingly not been in focus before. The stability of structures in the mandibular canine region during development is based on results from normal individuals (Kjær 1975, 1989, 2017) and this stability is a prerequisite for developing the canine axis method for defining misplacement.

In the mandibular canine region, developmental disorders in the primary and permanent dentitions have been described. Cases of mandibular primary dentitions with fusions between the primary canine and the primary lateral incisor have been investigated. In these cases, agenesis occurred of the permanent mandibular lateral incisor, but not of the permanent mandibular canine (Kjær and Daugaard-Jensen 2000). This study demonstrates the importance of the mandibular canine tooth bud for development of the canine region. The aetiology behind fusions is not known.

In the mandibular permanent dentitions, transpositions of the mandibular canine and the mandibular lateral incisor have been described and discussed from a genetic point of view (Peck et al. 1998). These types of transpositions might become clinically visible after eruption of a mandibular permanent canine where the crown originally was misplaced in a mesial position.

This study raises the question whether a misplaced tooth germ as demonstrated could be the aetiological background for ectopic mandibular canines.

Radiographs for diagnostics of permanent canine formation and eruption are very rarely taken in very young children. Radiographs of mandibular canines are normally taken in early puberty, if canine eruption seems to fail. This explains the ethical and clinical difficulties in early diagnosis of mandibular canine ectopia and explains why the question concerning initial positions of a permanent canine anlage has never been performed.

The similarities between the results in the present study and the comparing premolar study (Kjær 2021) are convincing. In both studies, the permanent tooth germ can start initial formation misplaced distally or mesially to the presiding primary tooth. Presumably, the space in the mandibular bone is decisive for the final position of the misplaced teeth. This is a factor not included in this present study.

The discrepancy between the results in the present study and the comparing premolar study (Kjær 2021) is that ankylosis in primary teeth with two roots seemingly plays a role in misplacement of the permanent tooth buds. This was seemingly not the case in the present study, where ankylosis was not observed in the one-rooted primary teeth.

The future perspective for a study like the present one is to be aware of malpositions of permanent crowns when first diagnosed. If such malpositions are followed up regularly, it might be possible to improve early diagnostics, prediction and eventually prevention and possibly also treatment of ectopic teeth. This study demonstrates and explains different malpositions of the developing crowns of the permanent mandibular canines.

## Conclusion

This study demonstrates and explains different malpositions of the developing crowns of the permanent mandibular canines. The results are considered of importance for future understanding of the aetiology behind ectopic mandibular canines.
